# Agonists for Bitter Taste Receptors T2R10 and T2R38 Attenuate LPS-Induced Permeability of the Pulmonary Endothelium *in vitro*

**DOI:** 10.3389/fphys.2022.794370

**Published:** 2022-03-23

**Authors:** Zsuzsanna Kertesz, Elizabeth O. Harrington, Julie Braza, Brianna D. Guarino, Havovi Chichger

**Affiliations:** ^1^Biomedical Research Group, Anglia Ruskin University, Cambridge, United Kingdom; ^2^Vascular Research Laboratory, Providence Veterans Affairs Medical Center, Providence, RI, United States; ^3^Department of Medicine, Alpert Medical School of Brown University, Providence, RI, United States

**Keywords:** ARDS, bitter taste receptors, lung endothelial cells, permeability, VE-cadherin, cAMP, Rac1

## Abstract

One of the hallmarks of acute respiratory distress syndrome (ARDS) is an excessive increase in pulmonary vascular permeability. In settings of ARDS, the loss of barrier integrity is mediated by cell–cell contact disassembly and actin remodelling. Studies into molecular mechanisms responsible for improving microvascular barrier function are therefore vital in the development of therapeutic targets for reducing vascular permeability seen in ARDS. Bitter taste receptors (T2Rs) belong to the superfamily of G-protein-coupled receptors found in several extraoral systems, including lung epithelial and smooth muscle cells. In the present study, we show for the first time that several T2Rs are expressed in human pulmonary arterial endothelial cells (HPAECs). Our results focus on those which are highly expressed as: T2R10, T2R14 and T2R38. Agonists for T2R10 (denatonium) and T2R38 (phenylthiourea), but not T2R14 (noscapine), significantly attenuated lipopolysaccharide (LPS)-induced permeability and VE-cadherin internalisation in HPAECs. In T2R10- or T2R38-siRNA knockdown cells, these endothelial-protective effects were abolished, indicating a direct effect of agonists in regulating barrier integrity. Our further findings indicate that T2R10 and T2R38 exert their barrier-protective function through cAMP but *via* Rac1-dependent and independent pathways, respectively. However, using an *in vivo* model of ARDS, the T2R38 agonist, phenylthiourea, was not able to protect against pulmonary edema formation. Taken together, these studies identify bitter taste sensing in the pulmonary endothelium to regulate barrier integrity *in vitro* through cAMP-Rac1 signalling.

## Introduction

Over 10% of patients in intensive care units worldwide suffer from acute respiratory distress syndrome (ARDS), associated with a mortality rate of nearly 40% (Rubenfeld et al., 2005; Matthay et al., 2019). The main predisposing factors which lead to ARDS are pneumonia, major surgery, trauma or sepsis (ARDS Definition Task Force et al., 2012) and, more recently, SARS-CoV2 (COVID-19) infection (Huang et al., 2020). Patients with ARDS suffer from acute hypoxemic respiratory failure, so mechanical ventilation is one of the key therapeutic approaches to improve hypoxemia; however, this treatment has been linked to worsening of the respiratory failure for some patients (ARDS Definition Task Force et al., 2012). One of the hallmarks of ARDS is an increase in lung endothelial permeability, associated with the development of pulmonary edema in these patients (Matthay et al., 2019). It is therefore vital to understand the mechanisms which regulate endothelial permeability, with the aim of reducing respiratory failure in patients with ARDS.

One of the key mechanisms which mediates vascular leak across the endothelial monolayer is the disruption of cell–cell contacts, maintained by the adherens junction complex, and an increases in actin-myosin contractility (Komarova and Malik, 2010; Kuppers et al., 2014; Trani and Dejana, 2015). In the case of the barrier-disruptive agent lipopolysaccharide (LPS), an endotoxin from Gram-negative bacteria, increased permeability is mediated through its binding to Toll-like receptor 4 (Aoki et al., 2015; Modhiran et al., 2015). The resulting pathway, through guanine exchange factor, Rho, Rho-associated coiled-coil containing protein kinase, rapidly accelerated fibrosarcoma and mitogen-activated protein kinase, destabilises endothelial junctions, through downregulation of intracellular 3′,5′-cyclic adenosine monophosphate (cAMP) levels and inactivation of the small Rho GTPase, Rac1, to cause myosin light chain (MLC) phosphorylation and vascular leak (Schlegel and Waschke, 2009; Haidari et al., 2012). Targeting these molecular mechanisms has been shown to attenuate LPS-induced pulmonary edema formation *in vivo* (Birukova et al., 2013; Barabutis et al., 2018; Wang et al., 2021) and therefore offers potential therapeutic value in treating patients with ARDS.

Previous studies demonstrate the expression of the G-protein-coupled sweet taste receptor, taste receptor type 1 member 3 (T1R3), in the pulmonary vasculature, with expression levels downregulated following exposure to LPS (Harrington et al., 2018). Exposure of lung microvascular endothelial cells to sucralose, an agonist of T1R3, attenuated LPS-induced endothelial barrier dysfunction. Likewise, sucralose exposure attenuated bacteria-induced lung edema formation *in vivo*. Inhibition of the sweet taste signalling pathway, through zinc sulphate, or siRNA for T1R3 or alpha gustducin, blunted the protective effects of sucralose on the endothelium. Further studies indicate the role of key endothelial signalling molecules, such as Src, p21-activated kinase (PAK) and MLC2, as regulated by T1R3 agonists. While this research demonstrates the importance of the sweet taste receptor signalling in the lung endothelium, there have been fewer such studies with other taste receptors.

The expression of bitter taste receptors (T2Rs) and their downstream signalling molecules have been found in several extraoral systems, including the digestive, respiratory and genitourinary systems, as well as in brain and immune cells (Lu et al., 2017; Conaway et al., 2020) where they carry out different biological functions tailored to their location. Research interest in taste receptors in the lung has focused on bitter taste receptors in the specialised airway epithelium (Shah et al., 2009) and smooth muscle (Deshpande et al., 2010) rather than the endothelium. More recently, T2R protein and *TAS2R* mRNA have also been identified in resident and tissue infiltrating immune cells in the lungs (Shah et al., 2009; Orsmark-Pietras et al., 2013; Ekoff et al., 2014; Lee et al., 2014). T2R agonists, including those identified in bacteria such as quinolones, cause relaxation of the airway smooth muscle and decreased airway resistance, as well as enhanced movement of motile cilia and increased phagocytosis (Deshpande et al., 2010; Sharma et al., 2016, 2017; Gopallawa et al., 2021). Therefore, T2R agonists are being further investigated as a potential therapeutic approach to reduce airway inflammation and hyperresponsiveness associated with allergic asthma. While immunofluorescence staining of human omental arteries suggests the presence of taste 2 receptor member 7 (T2R7) in the endothelium of mesenteric and omental arteries (Chen et al., 2017), the complete identification of T2R in the endothelium or the potential role in pulmonary function of these G-protein-coupled receptors (GPCRs) has not previously been studied.

In humans, 25 members of the T2R family have been identified to sense bitter taste agonists with high specificity to ensure high sensitivity of humans to bitter, and likely toxic, stimuli (Meyerhof et al., 2010; Raka et al., 2019). In the present study, we investigate the expression of T2Rs in a cell culture model of the human pulmonary endothelium, human pulmonary large vessel arterial endothelial cells (HPAEC). We further study the effect of specific T2R agonists on endothelial barrier function and establish the molecular mechanism through which T2Rs regulate the pulmonary endothelium.

## Materials and Methods

### Cell Lines and Reagents

HPAEC and media were purchased from ATCC (Teddington, United States). Cells were cultured in vascular cell media, supplemented with endothelial cell growth kit-BBE and 1% penicillin/streptomycin, and used between passage 3 and 8. Reagents for siRNA studies (siRNA, transfection reagent and scrambled control) were purchased from Dharmacon (Cambridge, United Kingdom). Antibodies for bitter taste receptors T2R10, T2R14 and T2R38 were obtained from Santa Cruz (Texas, United States), BosterBio (California, United States) and Novus Bio (Abingdon, United States), respectively, whereas the VE-cadherin antibody was purchased from Millipore (Watford, United Kingdom). The cAMP-Screen Direct System kit was purchased from Applied Biosystems (now Thermo Fisher, Paisley, United Kingdom) and the G-LISA^®^ Rac1 Activation Assay Biochem Kit^™^ was obtained from Cytoskeleton, Inc. (Colorado, United States). Human-specific primers for *TAS2R* (Table 1) were designed based on previously published studies (Upadhyaya et al., 2014) and purchased from Thermo Fisher Scientific (Paisley, UK), as were primers specific to human GADPH, β-actin and TBP and the TRIzol™ reagent. *Pseudomonas aeruginosa* strain PA103 was a kind gift from Dr. Troy Stevens (University of South Alabama, Mobile, AL). All other materials, including fluorescent secondary antibodies and LPS endotoxin from Escherichia coli serotype 0111:B4, were purchased from Sigma Aldrich/Merck (Dorset, United Kingdom).

**Table 1:** DNA primer sequences used to detect human T2R in HPAEC (based on Upadhyaya et al., 2014).

**Bitter taste receptor (*TAS2R*)**	**Gene accession ID**	**Primer sequence (5′–3′)**	
		**Forward**	**Reverse**
TAS2R1	NM_019599	TGTGGTGGTGAATGGCATTG	CAGCACTTACTGTGGAGGAGGAAC
TAS2R3	NM_016943	ACACATGATTCAGGGATAATAATGCAAA	TTAGCCATCTTGGTTTTTGGTAGGAAATT
TASR4	NM_016944	TACAGTGGTCAATTGCAAAACTTGG	AATGTCCTGGAGAGTAAAGGGTGG
TASR5	NM_018980	TGGTCCTCATATAACCTCATTATCCTGG	CTGCCATGAGTGTCTCCCA
TASR7	NM_023919	TGTTTTATATTGGTGCTATATCCAGATGTCTATGC	GGATAAATGAATGACTTGAGGGGTAGATTAGAG
TASR8	NM_023918	TTGATATGGTGGTGCACTGG	GTGAGTGACCCAAGGGGTAG
TASR9	NM_023917	TGAATTGACCATAGGGATTTGGG	ATAATTAGAATGAATGAATGGCTTGATGG
TASR10	NM_023921	GACTTGTAAACTGCATTGACTGTGCC	AAAGAGGCTTGCTTTAGCTTGCTG
TASR13	NM_023920	GGGTCAGTAAAAGAGAGCTGTCCTC	ATCAGAAGAAAGGAGTGGCTTGAAG
TASR14	NM_023922	GCTTTGGCAATCTCTCGAATTAGC	CTCTAAATTCTTTGTGACCTGAGGGC
TASR16	NM_016945	CCTGGGAATTTTTTAATATCCTTACATTCTGGT	GAAGCGCGCTTTCATGCTT
TASR38	NM_176817	ACAGTGATTGTGTGCTGCTG	GCTCTCCTCAACTTGGCATT
TASR39	NM_176881	TGTCGCCATTTCTCATCACCTTA	ATTGAGTGGCTGGCAGGGTAG
TASR40	NM_176882	AGAGTGCATCACTGGCATCCTT	GAGGATGAGAAAGTAGCTGGTGGC
TASR41	NM_176883	GGTTGCTGCCCTTGGATATGA	TGAAGATGAGGATGAAGGGATGG
TASR42	NM_181429	ATGGCCACCGAATTGGACA	GCTTGCTGTTTCCCAGAATGAG
TASR43	NM_176884	GGTCTCCAGAGTTGGTTTGC	TCTTGTTTCCCCAAATCAGG
TASR44	NM_176885	CATTGGTAAATTCCATTGAGC	GATATCATTATGGACAGAAAGTAAAC
TASR45	NM_176886	CTCCTTTGCTGACCAAATTGTC	GAACGGGTGGGCTGAAGAAC
TASR46	NM_176887	GAGTTGAATCCAGCTTTTAAC	ATAGCTGAATGCAATAGCTTC
TASR47	NM_001097643	GGTGTTATTACTACATTGGTATGCAACTC	AAGACAGGTTGCTTTTCCAGC
TASR48	NM_176888	GGTTTACTCTGGGTCATGTTATTC	TTTGCTCTGCTGTGTCCTAAG
TASR49	NM_176889	GCACTGATAAATTTCATTGCCTGG	TTGTTCCCCCAAATCAGAATGAA
TASR50	NM_176890	ATGTGGCTTGCTGCTAACCT	CAGCCTTGCTAACCATGACA
TASR60	NM_177437	CAGGCAATGGCTTCATCACTG	TCCCACACCCAGAATTTAAAGTCC
TBP	NM_003194.4	CAGCTTCGGAGAGTTCTGGG	GGGCACTTACAGAAGGGCAT
GAPDH	NM_002046	TGTGAGGAGGGGAGATTCAG	ACCCAGAAGACTGTGGATGG
β-actin	NM_001101.3	CACCAACTGGGACGACAT	ACAGCCTGGATAGCAACG

### mRNA Expression Analysis

mRNA analysis of *TAS2R* expression in HPAECs was performed using previously published primers specific to human *TAS2Rs* (Upadhyaya et al., 2014). Total RNA was extracted from HPAEC using the TRIzol^™^ reagent and following the manufacturer’s instructions. RNA was purified and reverse transcribed as previously described (Harrington et al., 2018; Lizunkova et al., 2019) and T2R transcripts were measured using PCR primers (Table 1). TBP was used as the housekeeping gene and analysis was verified with housekeeping genes β-actin and GAPDH. Relative gene expression level was analysed for each T2R using the ΔCt method where ΔCt = (Ct_TAS2R_ – Ct_TBP_) corresponding to the detected threshold cycles for the target and housekeeping gene.

### Whole Cell ELISA and Western Blotting

Cell surface expression of VE-cadherin and T2Rs (T2R10, T2R14 and T2R38) was measured using the whole cell ELISA as previously described (Chichger et al., 2015). In brief, HPAEC were treated with LPS in the presence and absence of T2R agonists noscapine, denatonium or phenylthiourea (0.1 mm) for 48 h. Studies were also performed with the addition of NSC-23766 (10 μm; Birukova et al., 2013) to inhibit Rac1 activity. Cells were then fixed using 1% paraformaldehyde at room temperature for 10 min. Whole cell ELISA was then performed using antibodies specific to the extracellular domain of VE-cadherin (BV6, amino acid unspecified; Su and Kowalczyk, 2017), T2R10 (D-12, amino acid unspecified, sc-169,473), T2R14 (Boster BioPA5-39,710, amino acid 229–278) and T2R38 (Novus, NBP2-33711), and 488-fluorescent-conjugated secondary antibodies measured at 1 s exposure time at 485/552 ex/em using a fluorescent plate reader (Victor, Perkin Elmer).

Whole cell expression of VE-cadherin and T2Rs (T2R10, T2R14 and T2R38) was performed using Western blotting and using T1R3, TBP and actin as reference. HPAEC were treated as for the whole cell ELISA and lysed with RIPA buffer, scraped over ice, centrifuged and resuspended in Laemmli buffer (50 μg). Samples were then subjected to immunoblot analysis with 10% SDS-PAGE and using primary antibodies specific to T2R10, T2R14, T2R38, T1R3 and reference proteins at a dilution of 1:1000, except TBP and actin (1,5,000), and secondary antibodies at dilutions of 1:5000. Densitometry was performed using gel analysis software on ImageJ.

### *In vitro* Permeability Assay

Endothelial monolayer permeability was measured using the fluorescein isothiocyanate (FITC)-dextran assay, as previously described (Lizunkova et al., 2019). HPAEC were seeded into Transwell inserts in a 24-well plate and cultured for 26 h, followed by exposure to LPS (1 μg/ml) and T2R agonists noscapine, denatonium or phenylthiourea (0.1 mm) for a further 10 h. At this time point, FITC-conjugated 4 kD dextran (1 mg/ml) was added to the upper chamber of the Transwell insert for 30 min. Media was then collected from the lower chamber of the Transwell insert (100 μl) and measured for FITC concentration at 485/535 ex/em using a fluorescent plate reader (Victor, Perkin Elmer). Permeability in relative fluorescence units was used to calculate % permeability as the fluorescence in the lower chamber divided by that in the upper chamber and multiplied by 100 and normalised to vehicle treatment.

### siRNA Transfection

For some experiments, HPAEC were transiently transfected with T2R10 and T2R38 SMARTpool siGENOME siRNA duplexes (100 nm), or non-specific (ns), scrambled duplexes, using the Dharmafect^™^ reagent 4, as per the manufacturer’s guidelines. At 42 h following transfection, cells were exposed to denatonium or phenylthiourea (0.1 mm) in the presence or absence of LPS (1 μg/ml) for a further 6 h and permeability, VE-cadherin surface expression, cAMP activity or Rac1 activity were measured. Confirmation of knockdown was performed using Western blotting and whole cell ELISA with antibodies specific to T2R10 or T2R38.

### cAMP and Rac1 Activity Assays

cAMP levels and Rac1 activity were measured using a cAMP-screen direct system assay kit and a Rac1 activation assay kit, respectively. For both assays, HPAEC were seeded and cultured for 42 h, followed by exposure to LPS (1 μg/ml) and T2R agonists noscapine, denatonium or phenylthiourea (0.1 mm) for a further 6 h. Alternatively, siRNA knockdown was performed followed by treatment. Cell lysates were then prepared and the assay kits were performed as per the manufacturer’s guidelines. For cAMP, levels were quantified as relative luminescence units from the CSPD^®^/Sapphire-II™ RTU substrate/enhancer solution and measured at 1 s exposure time using a plate reader (Victor, Perkin Elmer). For Rac1 activity, the absorbance of HRP-conjugate was measured at 490 nm optical density using a plate reader (SUNRISE, Tecan).

### *In vivo* Permeability Assay

Pulmonary edema was measured as wet-dry lung weights, as previously described (Harrington et al., 2018). Adult male 8–10 week old C57BL/6 mice were administered with bitter agonist, phenylthiourea (10 mg/kg) by retro-orbital injection or the vehicle control (H_2_O). At 44 h post-injection, live Gram-negative bacteria *P. aeruginosa* (PA103), or saline vehicle, administered *via* a single intratracheal injection (10^7^ colony-forming units) for 4 h. Mice were then euthanised with 1–4% isoflurane and lungs were excised for wet and dry weights. All animal experimental protocols were approved by the Institutional Animal Care and Use Committees of the Providence Veterans Affairs Medical Center and Brown University and comply with the Health Research Extension Act and the National Institutes of Health guidelines.

### Statistical Analysis

Data was analysed using GraphPad Prism 7 software and presented as mean ± standard deviation (SD) with significance denoted as *p* < 0.05. Sample size is 5–6 for *in vitro* studies and 7–8 for *in vivo* studies. Statistical analysis was performed using either an unpaired Student’s t-test, one-way or two-way ANOVA and, where relevant, a Tukey multiple comparison post-hoc test was performed.

## Results

### T2R10, T2R14 and T2R38 Are Highly Expressed in Human Pulmonary Arterial Endothelial Cells

To investigate whether T2Rs were expressed in pulmonary arterial endothelial cells, primers mapped to 25 human *TAS2R* were used (Table 1). mRNA expression of 16 *TAS2R* was undetected in HPAECs, whereas *TAS2R10*, *TAS2R14* and *TAS2R38* were highly expressed relative to the internal reference, TATA-box binding protein (*TBP*; Table 2). Further, mRNA expression of six *TAS2R* (*TAS2R1, TAS2R3, TAS2R4, TAS2R16, TAS2R40* and *TAS2R43*) was found to be expressed at lower levels (0.82- to 0.12-fold expression relative to *TBP*). Protein expression of T2R10, T2R14 and T2R38 was confirmed in cell lysate, using Western blotting, and at the cell surface, using whole cell ELISA, with VE-cadherin used as an internal reference. All three T2Rs were expressed at a protein level in HPAEC lysate and at the cell surface, with levels comparable to that seen for the sweet taste receptor (T1R3; Table 3). Taken together, these data demonstrate the mRNA expression of several *TAS2R*s and protein expression of T2R10, T2R14 and T2R38 in human pulmonary arterial endothelial cells.

**Table 2:** mRNA expression levels of bitter taste receptors in human pulmonary endothelial cells.

**Bitter taste receptor** (*TAS2R*)	**mRNA expression** (relative to TBP)
TAS2R38	2.43 ± 0.29
TAS2R14	2.41 ± 0.48
TAS2R10	2.09 ± 0.37
TBP (positive control)	1 (reference)
TAS2R4	0.82 ± 0.55
TAS2R16	0.52 ± 0.29
TAS2R1	0.49 ± 0.46
TAS2R3	0.49 ± 0.23
TAS2R40	0.35 ± 0.49
TAS2R43	0.12 ± 0.28

mRNA expression of TAS2R demonstrated as relative to the positive control (TBP) which was normalised as 1. Expression < 0.1 was considered too low to accurately demonstrate expression. *Data are presented as mean ± S.D, n=5-6*.

**Table 3:** Protein expression levels of bitter taste receptors in human pulmonary endothelial cells.

**Bitter taste receptor**	**Whole cell protein expression** (relative to VE-cadherin)	**Cell surface protein expression** (relative to VE-cadherin)
T2R38	0.56 ± 0.11	0.28 ± 0.04
T2R14	0.68 ± 0.09	0.16 ± 0.09
T2R10	0.22 ± 0.15	0.22 ± 0.03
VE-cadherin (positive control)	1 ± 0.30 (reference)	1 ± 0.11 (reference)
T1R3	0.51 ± 0.22	0.25 ± 0.06

*Cell surface and lysate protein expression was evaluated using whole cell ELISA and Western blot of cell lysate respectively. Data are presented as mean ± S.D, n=5-6*.

### Agonists for T2R10, T2R14 and T2R38 Differentially Regulate Pulmonary Barrier Function in an *in vitro* Model of ARDS

Previous studies have demonstrated a role for the sweet taste receptor in protecting the pulmonary endothelium against LPS-induced increase in permeability (Harrington et al., 2018). Our next set of experiments therefore assessed the effect of T2Rs in regulating endothelial barrier function using the documented agonists for T2R10, T2R14 and T2R38: denatonium, noscapine and phenylthiourea, respectively (Kim et al., 2003; Sainz et al., 2007; Meyerhof et al., 2010; Born et al., 2013).

**Figure 1:**
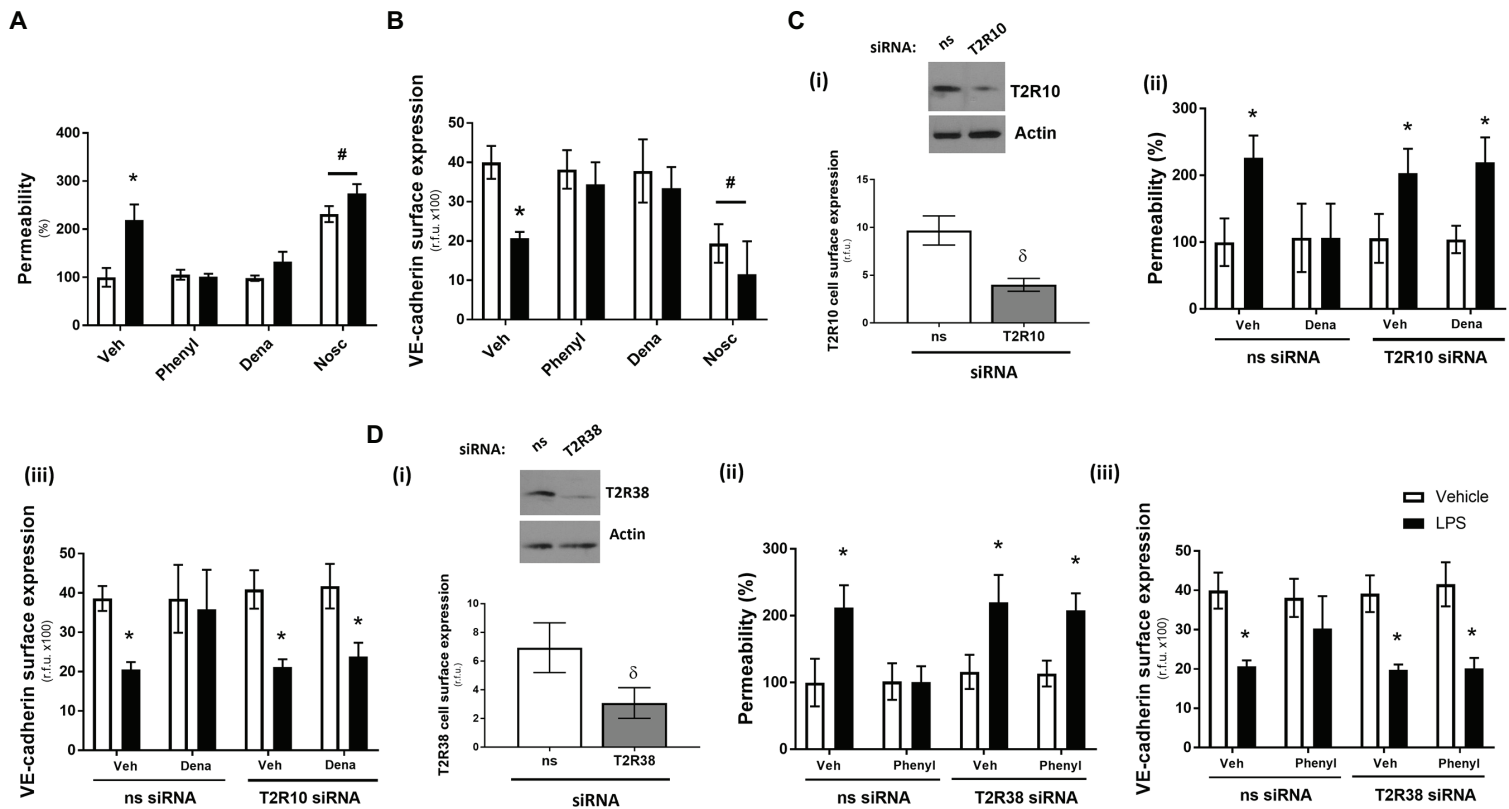
Denatonium (Dena) and phenylthiourea (Phenyl) protect against LPS-induced permeability and VE-cadherin internalisation in human pulmonary arterial endothelial cells by acting directly through T2R10 and T2R38, respectively. Panels A and B: HPAEC were exposed to noscapine, denatonium or phenylthiourea (0.1 mm) in the presence (closed bars) or absence (open bars) of LPS (1 μg/ml) for 24 h. Monolayer permeability was measured using the FITC-dextran essay (panel **A**) and VE-cadherin surface expression was assessed using whole cell ELISA (panel **B**). Panels C and D: T2R10 (panel **C**)- and T2R38 (panel **D**)-specific siRNA was used to knockdown expression in HPAEC and confirmed using Western blot (inset) and whole cell ELISA [panels **C**(i) and **D**(i)]. Denatonium or phenylthiourea (0.1 mm) was added after 24 h in the presence (closed bars) or absence (open bars) of LPS (1 μg/ml) and monolayer permeability [panels **C**(ii) and **D**(ii)] and VE-cadherin surface expression [panels **C**(iii) and **D**(iii)] were studied. Data are presented as mean ± S.D. *n* = 6. Panel ^*^p < 0.05 versus vehicle for LPS; ^#^*p* < 0.05 versus vehicle for bitter agonists; ^δ^*p* < 0.05 versus non-specific siRNA.

Using the Transwell permeability assay, in the absence of LPS, agonists for T2R10 (denatonium) and T2R38 (phenylthiourea) had no effect on endothelial barrier function of HPAECs while the agonist for T2R14 (noscapine) significantly increased baseline permeability (Figure 1A). Following exposure to LPS, phenylthiourea and denatonium blunted endothelial monolayer permeability, whereas noscapine exacerbated LPS-induced leak (Figure 1A). These findings were mirrored in measurement of VE-cadherin cell surface expression using the whole cell ELISA, with phenylthiourea and denatonium preserving VE-cadherin surface expression in the presence of LPS and noscapine resulting in significantly reduced VE-cadherin surface levels (Figure 1B).

To establish that the T2R agonists phenylthiourea and denatonium are regulating barrier function through T2R38 and T2R10, respectively, siRNA knockdown studies in HPAECs were performed with each taste receptor. A significant reduction in protein expression of T2R10 and T2R38, following siRNA knockdown, was confirmed in cell lysate (Western blot) and at the cell surface (whole cell ELISA) compared to the non-specific siRNA control [Figures 1C(i), D(i)]. Interestingly, knockdown of each receptor, in the absence of taste agonist, did not impact baseline or LPS-induced monolayer permeability [Figures 1C(ii), D(ii)] or VE-cadherin surface expression [Figures 1C(iii), D(iii)]. In the presence of denatonium, endothelial cells transfected with T2R10 siRNA were unable to protect against LPS-induced barrier leak [Figure 1C(ii)] or VE-cadherin internalisation [Figure 1C(iii)]. Likewise, following exposure to phenylthiourea, cells transfected with T2R38 siRNA were unable to protect the endothelium against LPS-induced barrier disruption [Figure 1D(ii).

Taken together, these data show that bitter taste agonists, phenylthiourea and denatonium, protect the pulmonary endothelium against LPS-induced barrier disruption, through T2R38 and T2R10, respectively.

### T2R38, but Not T2R10, Agonist Protects Against LPS-Induced Barrier Disruption Through Preservation of Rac1 Activity

LPS has been observed to reduce cAMP and Rac1 expression in endothelial cells resulting in barrier leak (Schlegel and Waschke, 2009). Therefore, to understand the molecular mechanism through which denatonium and phenylthiourea exert barrier-protective effects on human pulmonary arterial endothelial cells, these signalling molecules were studied.

**Figure 2:**
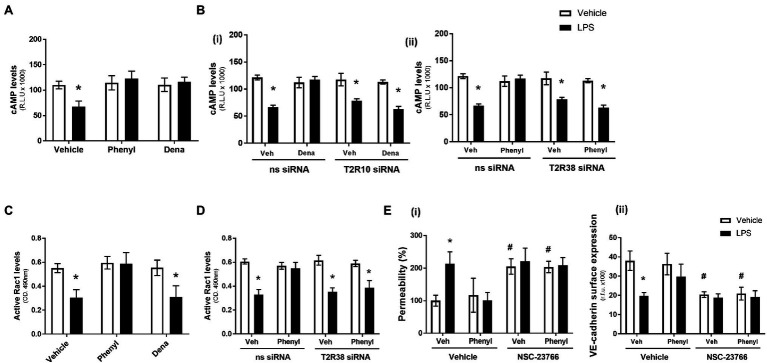
Phenylthiourea (Phenyl), but not denatonium (dena), acts through a cAMP-Rac1-dependent signalling pathway to protect the pulmonary endothelial barrier. Panel **A** and **C**: HPAEC were exposed to noscapine, denatonium or phenylthiourea (0.1 mm) in the presence (closed bars) or absence (open bars) of LPS (1 μg/ml) for 24 h. cAMP (panel **A**) and Rac1 (panel **C**) levels were measured using commercially available ELISA kits. Panel **B** and **D**: T2R10 [panel **B**(i)]- or T2R38 [panel **B**(ii)]-specific siRNA was used to knockdown expression in HPAEC and denatonium or phenylthiourea (0.1 mm), respectively, was added after 24 h in the presence (closed bars) or absence (open bars) of LPS (1 μg/ml). cAMP (panel **B**) and Rac1 (panel **D**) levels were measured using commercially available ELISA kits. Panel **E**: HPAEC were exposed to the Rac1 inhibitor, NSC-23766, with phenylthiourea (0.1 mm) in the presence (closed bars) or absence (open bars) of LPS (1 μg/ml) for 24 h. Monolayer permeability was measured using the FITC-dextran essay [panel **E**(i)] and VE-cadherin surface expression was assessed using whole cell ELISA [panel **E**(ii)]. Data are presented as mean ± S.D. *n* = 5, 6. Panel ^*^p < 0.05 versus vehicle for LPS; ^#^*p* < 0.05 versus vehicle for NSC-23766.

In the absence of LPS, the T2R10 and T2R38 agonists, denatonium and phenylthiourea, had no effect on cAMP levels (Figure 2A). As anticipated, exposure of HPAEC to LPS significantly decreased cAMP levels but, interestingly, phenylthiourea and denatonium attenuated the LPS-induced reduction in cAMP levels to preserve the signalling molecule (Figure 2A). Further study using HPAEC transfected with T2R10 [Figure 2B(i)] or T2R38 [Figure 2B(ii)] siRNA demonstrates that each agonist preserves cAMP levels through their respective bitter taste receptor. That is, siRNA knockdown of T2R10 blocks the protective effect of denatonium in preserving cAMP levels [Figure 2B(i)] and T2R38 knockdown attenuates the ability of phenylthiourea to maintain cAMP expression in endothelial cells [Figure 2B(ii)]. These studies indicate that both denatonium and phenylthiourea are dependent on their respective T2R to protect against LPS-induced loss of cAMP levels.

Similar to cAMP levels, in the absence of LPS, denatonium and phenylthiourea had no impact on Rac1 activity levels while LPS exposure, as expected, significantly reduced Rac1 activity (Figure 2C). In contrast, however, in the presence of LPS, only phenylthiourea was able to maintain Rac1 activity levels (Figure 2C). siRNA knockdown of T2R38 significantly blunted the ability of phenylthiourea to protect against LPS-induced loss of Rac1 activity (Figure 2D), indicating that phenylthiourea preserves Rac1 activity, in settings of LPS injury, through T2R38.

The role of Rac1 in mediating the barrier-protective effect of phenylthiourea was investigated next using the established Rac1 inhibitor, NSC-23766 (Birukova et al., 2013). Exposure of HPAEC to the Rac1 inhibitor, in the absence of LPS, significantly increased endothelial barrier leak [Figure 2E(i)] and reduced VE-cadherin surface expression [Figure 2E(ii)]. Interestingly, when exposed to the Rac1 inhibitor, phenylthiourea was unable to attenuate LPS-induced barrier disruption [Figure 2E(i)] or VE-cadherin internalisation [Figure 2E(ii)]. These data indicate that phenylthiourea protects the endothelium against LPS-induced injury through preserving Rac1 activity.

### Phenylthiourea Does Not Protect the Pulmonary Endothelium *in vivo*

In the last set of experiments, the barrier-protective effect of phenylthiourea was assessed using an established *in vivo* model of endothelial barrier leak, live *P. Aeruginosa* bacteria (PA103; Harrington et al., 2018). Lung wet to dry ratio was assessed following retro-orbital injection with the T2R38 agonist in the presence and absence of *P.* Aeruginosa. In the absence of bacteria exposure, phenylthiourea had no impact on wet-dry ratio (vehicle: 4.90 ± 0.18; phenylthiourea: 4.84 ± 0.29; *p* > 0.05; *n* = 7, 8). Exposure to *P. Aeruginosa* resulted in a significant increase in lung wet-dry weight ratio from 4.90 ± 0.18 (*n* = 8) to 6.63 ± 0.64 (*n* = 7), *p* < 0.05. Rather than protecting the pulmonary endothelium against *P. Aeruginosa,* phenylthiourea exposure exacerbated bacteria-induced increase in lung wet-dry ratio from 6.63 ± 0.64 (*n* = 7) to 7.55 ± 0.57 (*n* = 7), *p* < 0.05. These data demonstrate that the *in vitro* protection exerted by phenylthiourea, against endotoxin-induced leak across the pulmonary endothelial barrier, was not observed using an *in vivo* bacterial model of leak in the lungs.

## Discussion

In this study, we identify, for the first time, the presence of bitter taste receptors in human pulmonary arterial endothelial cells. We further demonstrate that agonists for two highly expressed receptors (T2R10 and T2R38) protect the pulmonary endothelium against LPS-induced barrier disruption and confirm that this protective mechanism is directly through the taste receptors. The findings here observe that denatonium, the agonist for T2R10, protects the endothelium through a cAMP-dependent, Rac1-independent mechanism while, phenylthiourea, the agonist for T2R38, attenuates LPS-induced barrier disruption in a cAMP/Rac1-dependent pathway. Finally, we demonstrate that this *in vitro* protection is not observed using an *in vivo* model of pulmonary endothelium leak.

T2Rs are a family of G-protein-coupled receptors (GPCRs) which consist of at least 25 receptor subtypes (Adler et al., 2000; Chandrashekar et al., 2000). In recent years, T2Rs have been localised to several extraoral systems where they exert different functions depending on their locations (Lu et al., 2017; Conaway et al., 2020). For example, agonists for T2R38 can increase the ciliary beat frequency in human airway epithelia (Lee et al., 2016) while T2R10 agonists attenuated airway hyperresponsiveness in airway smooth muscle, indicating the potential therapeutic value of T2Rs in the treatment of obstructive airway diseases (Shah et al., 2009; Deshpande et al., 2010). Interestingly, a recent deep sequencing study with COVID-19 patients admitted to the Intensive Care Unit shows significant alternative transcription differences in *TAS2R14*. Patients who died from COVID-19 showed 0.15892% splice variant versus 0.03625% in patients with COVID-19 who lived (Monaghan et al., 2021) suggesting a role for the receptor in injury. Although previous work has indicated the effect of bitter taste agonists, naringenin and denatonium, in elevating neovascularisation in tumours (Dmytrenko et al., 2017), no studies have assessed the presence or activity of T2Rs in human pulmonary endothelial cells. Our study identifies different levels of mRNA expression of various T2Rs on HPAEC and confirms the protein expressions of T2R10, T2R14 and T2R38 at similar levels as the sweet taste receptor, T1R3, as reported previously (Harrington et al., 2018). We have shown here that T2R10 and T2R38 agonists, denatonium and phenylthiourea, respectively, also have protective effects on the barrier function of endothelium as they were able to attenuate the LPS-induced permeability and VE-cadherin expression on HPAEC. These studies indicate that these bitter taste agonists have the potential to reduce pulmonary vascular leak; however, *in vivo* studies show that phenylthiourea, when administered retro-orbitally, exacerbates *Pseudomonas*-induced edema formation. This may be linked to the non-taste related toxic effects of phenylthiourea on the lung which outweigh the protective effect of activating T2R38 (Scott et al., 1990). Alternatively, exposure to taste molecules *via* retro-orbital-injection may exert a different response to those administered *via* oral gavage, as done for previous studies with sucralose (Harrington et al., 2018). Furthermore, *in vitro* studies presented were performed using human pulmonary arterial endothelial cells, whereas there is considerable endothelial cell heterogeneity in the lung (Aird, 2007a; Aird, 2007b; Comhair et al., 2012). Specifically, pulmonary microvascular endothelial cells, like pulmonary arterial endothelial cells, are often linked to fluid leak (Stevens et al., 2008). It is therefore possible that our findings in human pulmonary arterial endothelial cells do not fully mimic the pulmonary endothelium in physiological settings. Therefore, further studies should focus on studying the effect of T2R10 and T2R38 agonists in pulmonary microvascular endothelial cells, ideally those isolated from ARDS patients or a murine model of ARDS. In addition to vascular heterogeneity in the lung, there are several other cell types which have been shown to express T2Rs, including ciliated epithelium and both resident and lung infiltrating immune cells (Orsmark-Pietras et al., 2013; Ekoff et al., 2014). Therefore, while phenylthiourea may have a barrier-protective effect *in vitro*, the T2R38 selective bitter agonist may stimulate pro-edemagenic signals through epithelial and immune cells resulting in a lack of protection against *Pseudomonas*-induced edema formation *in vivo*. Investigation of the *Pseudomonas*-treated lung, following exposure to phenylthiourea, should be expanded to consider cell and protein composition of broncho-alveolar lavage fluid (BALF) and lung histology. Further studies could also focus on molecular activation of T2R38, as well as the route of instillation of the agonist, in pulmonary edema models *in vivo* or design of a selective T2R38 activator which lacks lung toxic effects.

LPS-induced endothelial barrier dysfunction is widely used as an experimental model for ARDS and characterised by increased monolayer permeability, disruption of intercellular junctions, increased actin-myosin contractility and edema formation (Liu et al., 2015). cAMP signalling has long been known to regulate the integrity of the endothelial barrier, strengthen cell–cell adhesions and increase endothelial barrier integrity (Birukova et al., 2013; Vassiliou et al., 2020). Studies using pulmonary endothelial cells show that the increase of intracellular cAMP levels enhances endothelial barrier properties and attenuates LPS-induced endothelial barrier dysfunction (Bogatcheva et al., 2009). Small guanosine triphosphatases (GTPases) from the Ras superfamily, primarily Rho GTPases (RhoA, Rac1 and Cdc42) or Rap1, have been shown to regulate cell adhesion and control permeability (Liu et al., 2015). It has been shown previously that iloprost improved LPS-induced endothelial barrier dysfunction in HPAEC could be suppressed by a Rac1 inhibitor, NSC-23766 (Birukova et al., 2013). In the present study, we investigated the role of cAMP and Rac1 in regulating the molecular mechanism through which T2R10 and T2R38 agonists mediate endothelial barrier protection. We found that agonists for T2R10 and T2R38 attenuated LPS-induced reduction of cAMP levels but only the T2R38 agonist was effective in blocking LPS-induced decrease in Rac1 levels. Furthermore, inhibition of Rac1 attenuated the barrier-protective effect of phenylthiourea. These results implicate the involvement of the cAMP/Rac1 pathway in the endothelial-protective effects of bitter taste receptors but the differential effects of signalling in T2R10 versus T2R38.

The current study focused on the most highly expressed T2Rs in HPAEC; however, the involvement of other T2Rs expressed (T2R1, T2R3, T2R4, T2R16, T2R40 and T2R43) in regulating endothelial barrier function was not explored. This should be the focus of further studies; however, future work with these receptors identified in HPAEC will need to consider the fact that T2Rs can recognise a repertoire of agonists which often overlap. For example, denatonium activates not only T2R10 but also T2R4 and T2R43 (Meyerhof et al., 2010). To date, a handful of T2R antagonists from different sources, mainly plants, have been found and they could also aid in the dissection of the different functions of T2R in HPAEC function.

## Data Availability Statement

The raw data supporting the conclusions of this article will be made available by the authors, without undue reservation.

## Ethics Statement

The animal study was reviewed and approved by Institutional Animal Care and Use Committees of the Providence Veterans Affairs Medical Center and Brown University to comply with the Health Research Extension Act and the National Institutes of Health guidelines.

## Author Contributions

EH and HC: study conception and design, data acquisition, analysis and interpretation, drafting and revision of the manuscript, accountability for accuracy and integrity of the data, and revision and approval of the manuscript. ZK and JB: data acquisition, analysis and interpretation, drafting and revision of the manuscript, and revision and approval of the manuscript. BG: revision of the manuscript and approval of the manuscript. All authors contributed to the article and approved the submitted version.

## Funding

This material is the result of work supported with resources and the use of facilities at Anglia Ruskin University and the Providence VA Medical Center. The work is supported with National Institutes of Health grants R01 HL123965 and P20 GM103652 (EH) and T32 HL134625 (EH and BG).

## Conflict of Interest

The authors declare that the research was conducted in the absence of any commercial or financial relationships that could be construed as a potential conflict of interest.

## Publisher’s Note

All claims expressed in this article are solely those of the authors and do not necessarily represent those of their affiliated organizations, or those of the publisher, the editors and the reviewers. Any product that may be evaluated in this article, or claim that may be made by its manufacturer, is not guaranteed or endorsed by the publisher.
